# Playback Theatre as a tool to enhance communication in medical education

**DOI:** 10.3402/meo.v18i0.22622

**Published:** 2013-12-23

**Authors:** Ramiro Salas, Kenya Steele, Amy Lin, Claire Loe, Leslie Gauna, Paymaan Jafar-Nejad

**Affiliations:** 1 Menninger Department of Psychiatry and Behavioral Sciences, Baylor College of Medicine, Houston, TX, USA; 2 Department of Family and Community Medicine, Baylor College of Medicine, Houston, TX, USA; 3 Houston Playback Theatre, Houston, TX; 4 School of Biomedical Informatics, University of Texas Health Science Center at Houston, Houston, TX, USA; 5 Department of Curriculum & Instruction, College of Education, University of Houston, Houston, TX, USA; 6 Jan and Dan Duncan Neurological Research Institute, Department of Molecular and Human Genetics, Baylor College of Medicine, Houston, TX, USA

**Keywords:** Playback Theatre, medical education, stress, improvisation, arts

## Abstract

Playback Theatre (PT) is an improvisational form of theatre in which a group of actors “play back” real life stories told by audience members. In PT, a conductor elicits moments, feelings and stories from audience members, and conducts mini-interviews with those who volunteer a moment of their lives to be re-enacted or “played” for the audience. A musician plays music according to the theme of each story, and 4-5 actors listen to the interview and perform the story that has just been told. PT has been used in a large number of settings as a tool to share stories in an artistic manner. Despite its similarities to psychodrama, PT does not claim to be a form of therapy.

We offered two PT performances to first year medical students at Baylor College of Medicine in Houston, Texas, to bring the students a safe and fun environment, conducive to sharing feelings and moments related to being a medical student. Through the moments and stories shared by students, we conclude that there is an enormous need in this population for opportunities to communicate the many emotions associated with medical school and with healthcare-related personal experiences, such as anxiety, pride, or anger. PT proved a powerful tool to help students communicate.

The first year of medical school is both a daunting and a defining time for future physicians. In the United States, many medical programs tend to be somewhat sequestered, high-pressure environments (1, 2) that allow students only limited time for “extracurricular” activities to offset the demands of professional training. Toward this end, we offered first-year students at Baylor College of Medicine the opportunity to participate in Playback Theatre (PT) performances in which they could share personal stories and experiences.

The PT activities were offered as part of Compassion and the Art of Medicine, a first-year elective course at Baylor that explores the roles of art in the healing process and the development of compassion in the medical education setting. The typical course enrollment is 50–75 students, and topics discussed include 1) international health, 2) patients’ or family members’ experiences with illness and doctors, 3) medical education as an art form, 4) compassionate physician role models and their experiences, and 5) balance of career with personal and family life.

In this setting, we sought to explore PT as a means of enhancing communication among students. We believe PT is intrinsically different from other extracurricular stress-reducing activities (e.g., playing sports or singing in a choir), as the primary goal is to facilitate the sharing (and receiving) of personally relevant, real-life experiences.

Playback Theatre was created in the 1970s by Jonathan Fox, a therapist and theatre performer in New York, as a way to explore personal stories through the art of theatre (3). In most improvisational theatres, the goal is to provoke laughter through witty remarks and physicality, often using offensive or pejorative remarks to create humorous situations. In PT, the goal is the opposite: The troupe works to create an environment based on trust where everyone feels safe and comfortable enough to tell a story from their lives – honoring and respecting tellers’ experiences no matter how silly, sad, joyful, or embarrassing they may be. When done successfully, this creates a channel of communication among PT audience members on a level that is difficult to achieve by any other means.

Houston Playback Theatre (HPT) was formed in 1996 and has performed in a wide array of venues – such as schools, churches, community centers, museums, and theatres. The performance themes have ranged from the most sensitive (e.g., stories from Alzheimer's patients and their relatives, cancer survival, and death) to the relatively light (e.g., stories of summer vacations). Over two years, HPT has provided two one-hour performances for the Compassion and the Art of Medicine course.

PT performances are designed and structured to ease the audience into sharing and thoughtful participation. At the beginning of a performance, actors introduce themselves by sharing moments from their lives – which are then immediately “played back” by the other actors. This models what is expected from the audience, and the tone of the performance is set by these introductions.

For the Compassion and the Art of Medicine performances, the HPT actors introduce themselves with personal experiences related to medicine. In PT, it is crucial that performers form “connections” with the audience; thus, it is helpful that several of the HPT actors are involved in health care as researchers, providers, or educators. As a result, it was possible to have a broad range of introductory moments that helped the actors bond with the audience of medical students.

After introductions, a PT performance moves to “short forms” in which the conductor elicits moments or feelings from audience members, and the actors play these on the spot, using various improvisational techniques. The third and usually major part of a PT performance is “stories.” Once audience members have warmed up to the idea of sharing a personal story, volunteers from the audience are invited to come to the stage, sit by the conductor, and tell their stories. After a brief interview, the conductor transitions the focus to the actors, who play the story on the spot. A typical performance has 3–4 stories told by different volunteers.

Lastly, the PT performance closes with a sharing of feelings elicited by the performance. This tends to reinforce the commonality of life experience and gives everyone time to reflect on the themes of the performance. Audience members often feel that the stories shared onstage resonate with their own stories, even if they did not volunteer to share theirs aloud during the performance.

In PT, the success of a performance is reflected in the range of stories told by the audience. Although funny stories are appreciated, a truly successful PT performance should include stories that elicit various emotions. In a medical education context, we expect PT to have three positive effects: First, it should offer a meaningful but entertaining experience, filled with laughter and emotion, which, in and of itself, is a welcome break from the stresses of academic study. Second, reflecting upon life through art should add meaning to personal stories – such as enriching or reinforcing students’ motivations to pursue medical careers (see comments 1 and 2, below). Lastly, telling personal stories could be therapeutic, as demonstrated in written and oral recounts of past traumatic events (4).

As a cursory means of exploring the positive impacts of communication among medical students, we reviewed the stories shared – as well as students’ reactions to the performance. From this, is seemed apparent that PT offered a safe place to freely share personal experiences – something visibly lacking in the medical curriculum. Below are selected “moments” shared by first-year medical students:

1. One student told about her first stethoscope experience – and the feeling of “Now I am a real doctor” once she clearly identified a heartbeat.
2. Another student “responded” with his own first stethoscope experience: not being able to find a pulse. The ensuing enactment was humorous, but it relayed an underlying sentiment of failure that the actors captured and played back.
3. A student told of witnessing a physician listing an array of physical problems to an elderly woman (e.g., her knee arthritis, other test results, etc.), who responded by repeatedly complaining of her hair loss – suggesting that the doctor was not really “listening” and was downplaying the patients’ concern for her changing image.
4. A student recounted how her grandfather was so proud about her being a medical student that he changed his mind about being buried – deciding instead (despite some religious doubts) to bequeath his body to a medical school. Her grandfather's decision, in turn, gave her an added respect for the cadavers in the dissection lab. She was better able to “humanize” who this person may have been, including his or her stories and the fact that someone must have loved him or her.
5. A student told of a trip to his home country when he was 10 years old, including feeling sick and experiencing a different health care system. Despite a humorous undertone, it relayed the importance of family heritage to him. More importantly, the story illustrated cultural differences in the health care system, but also similarities in the caring attitudes of medical personnel.
6. One student shared how she had conflicting emotions about becoming a doctor: She was very excited but was forced to leave her job as a teacher – where she was making a difference in the community. So, when the time comes to apply for off-site rotations, she planned to request placement in this same community. After the performance, an academic administrator confessed to one of the actors, “When that student shared this moment, I saw her in a new light. I didn't know she was a teacher before. I always try to place students where they request to be, but I can see now why being in that particular community would be especially important to her.”

The following are comments from students who attended the HPT performances:

1. “The performers of this lecture showed me that the means of healing or of encouraging healing does not necessarily need to be solely derived from pills and ointments, but can also incorporate the arts.”
2. “I gained some insights into how some non-medicinal practices could have a significant impact on the well-being of an individual, and possibly on the doctor–patient relationship.”
3. “It was absolutely amazing to see the actors, directed only by spoken words, accurately capture the feelings embedded in each story.”

## Conclusions

The stories freely shared by participating audience members, we believe, reflected a welcomed enhancement in both reflection and personal communication among these students. Given the high-stress environment in which medical students are immersed, PT shows promise in easing some of the pressure – and providing a semblance of balance to students’ lives. By sharing their experiences, students may feel less isolated and better able to recognize their reactions and feelings in others. Perhaps more importantly, PT provides a venue for the nourishment of a student community and, specifically, a student community based on mutual respect.

Strategic placement of PT performances within the medical curricula might foster students’ compassion and understanding. For example, an emerging part of modern medical training is early clinical experience – including empathy, an understanding of the impact of disease on patients, more confidence when talking to patients, and the development of communication skills (5). We believe that PT may offer a way to increase the value of those experiences by providing a structured way to discuss these topics.

Lastly, sharing a personal experience and witnessing others’ experiences through art can solidify meaning in a manner that is completely contrary to the memorization of “factoids.” Thus, PT could become a mechanism to aid in medical students’ professional identity development. Although we believe that offering such opportunities early is important, perhaps having a PT experience closer to graduation would be equally beneficial to revisit and reinforce students’ personal values and beliefs about medicine.
